# Intra‐colony spatial variance of oxyregulation and hypoxic thresholds for key *Acropora* coral species

**DOI:** 10.1002/ece3.11100

**Published:** 2024-03-05

**Authors:** Nicole J. Dilernia, Stephen Woodcock, Emma F. Camp, David J. Hughes, Michael Kühl, David J. Suggett

**Affiliations:** ^1^ Climate Change Cluster University of Technology Sydney (UTS) Ultimo New South Wales Australia; ^2^ School of Mathematical and Physical Sciences University of Technology Sydney (UTS) Ultimo New South Wales Australia; ^3^ National Sea Simulator Australian Institute of Marine Science (AIMS) Townsville Queensland Australia; ^4^ Department of Biology, Marine Biological Section University of Copenhagen Helsingør Denmark; ^5^ KAUST Reefscape Restoration Initiative (KRRI) and Red Sea Research Center (RSRC) King Abdullah University of Science and Technology Thuwal Saudi Arabia

**Keywords:** Coral oxyregulator, Coral reefs, Climate change, Hypoxic tolerance, Hypoxia Response Curves, Ocean deoxygenation

## Abstract

Oxygen (O_2_) availability is essential for healthy coral reef functioning, yet how continued loss of dissolved O_2_ via ocean deoxygenation impacts performance of reef building corals remains unclear. Here, we examine how intra‐colony spatial geometry of important Great Barrier Reef (GBR) coral species *Acropora* may influence variation in hypoxic thresholds for upregulation, to better understand capacity to tolerate future reductions in O_2_ availability. We first evaluate the application of more streamlined models used to parameterise Hypoxia Response Curve data, models that have been used historically to identify variable oxyregulatory capacity. Using closed‐system respirometry to analyse O_2_ drawdown rate, we show that a two‐parameter model returns similar outputs as previous 12th‐order models for descriptive statistics such as the average oxyregulation capacity (T_pos_) and the ambient O_2_ level at which the coral exerts maximum regulation effort (P_cmax_), for diverse *Acropora* species. Following an experiment to evaluate whether stress induced by coral fragmentation for respirometry affected O_2_ drawdown rate, we subsequently identify differences in hypoxic response for the interior and exterior colony locations for the species *Acropora abrotanoides*, *Acropora* cf. *microphthalma* and *Acropora elseyi*. Average regulation capacity across species was greater (0.78–1.03 ± SE 0.08) at the colony interior compared with exterior (0.60–0.85 ± SE 0.08). Moreover, P_cmax_ occurred at relatively low *p*O_2_ of <30% (±1.24; SE) air saturation for all species, across the colony. When compared against ambient O_2_ availability, these factors corresponded to differences in mean intra‐colony oxyregulation, suggesting that lower variation in dissolved O_2_ corresponds with higher capacity for oxyregulation. Collectively, our data show that intra‐colony spatial variation affects coral oxyregulation hypoxic thresholds, potentially driving differences in *Acropora* oxyregulatory capacity.

## Graphical abstract

We examine how intra‐colony spatial variance impacts coral hypoxic thresholds for common reef‐building *Acropora* species, providing new insights into coral hypoxia response curve analysis and oxyregulation capacity under ocean deoxygenation.

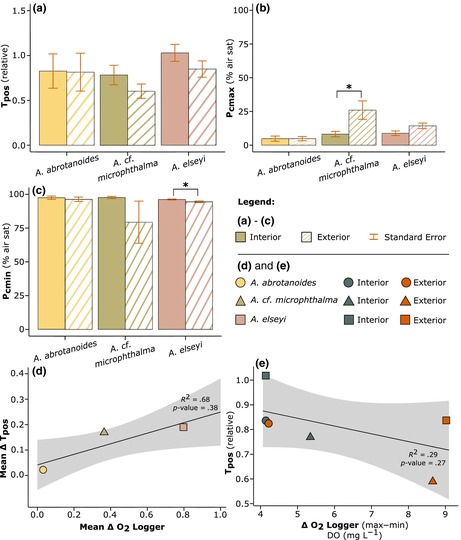

## 1 INTRODUCTION

Oxygen (O_2_) availability in marine systems (Breitburg et al., 2018), including coral reefs (Altieri et al., 2017; Hughes et al., 2020), is deteriorating under intensifying anthropogenic pressures. Loss of soluble O_2_ via ocean warming (Keeling et al., 2010; Sampaio et al., 2021) and increased biological O_2_ demand under reduced water quality (Keeling et al., 2010; Levin & Breitburg, 2015) can lead to deoxygenation—an often overlooked and relatively unstudied stressor on coral reefs (Hughes et al., 2020; Nelson & Altieri, 2019; Sampaio et al., 2021). Future global ocean projections under climate change predict deoxygenation of up to 7% by 2100 (Alderdice et al., 2021; Breitburg et al., 2018; Keeling et al., 2010; Pezner et al., 2023), and low O_2_ stress termed ‘hypoxia’ (<2 mg O_2_ L^−1^ or ~25% air sat) thus poses an increasing threat to coral reef health and survival (Altieri et al., 2017; Diaz & Rosenberg, 1995; Klein et al., 2020; Steckbauer et al., 2011; Vaquer‐Sunyer & Duarte, 2008). Whilst the full capacity for coral survival under increasing hypoxia remains unknown (Hughes, Alexander, et al., 2022; Johnson, Scott, et al., 2021), many marine organisms are being pushed to their lower limits for healthy metabolic functioning (Diaz & Rosenberg, 1995; Steckbauer et al., 2020; Vaquer‐Sunyer & Duarte, 2008), including cnidarians adapting to reduce metabolic rates (Rutherford & Thuesen, 2005; Vaquer‐Sunyer & Duarte, 2008), or relying on alternative sources of energy to survive under sustained hypoxic conditions (Linsmayer et al., 2020; Murphy & Richmond, 2016).

Hypoxia response curves (HRCs) are commonly employed to assess the performance of aquatic organisms (Carey et al., 2013; Cobbs & Alexander, 2018; Tremblay et al., 2020), and more recently corals (Hughes, Alexander, et al., 2022), under increasing hypoxia. Although hypoxic tolerance can be examined using various time versus dose‐dependent approaches (Alderdice et al., 2021; Alva García et al., 2022; Johnson, Scott, et al., 2021), the use of closed‐system respirometry for quantifying hypoxic response (or ‘performance’) curves—much like rapid thermal performance curves (e.g., Aichelman et al., 2019; Dilernia et al., 2023)—enable relatively high throughput. Hypoxia response curves display patterns of O_2_ consumption, that is, respiration (VO_2_, mg h^−1^) over time, against ambient levels of O_2_ (*p*O_2_, % air saturation) (Hughes, Alexander, et al., 2022). Historically, many organisms such as cnidarians (including corals) were assumed to exhibit respiration rates that varied in direct proportion to their ambient O_2_ level (Seibel et al., 2021; Shick, 1990; Ultsch & Regan, 2019)—an attribute known as oxyconformity (Hughes et al., 2020; Hughes, Alexander, et al., 2022). However, HRC measurements recently revealed that corals can in fact oxyregulate, controlling their respiration capacity irrespective of the ambient O_2_ (Hughes, Alexander, et al., 2022). Whilst Hughes, Alexander, et al. (2022) only studied a handful of coral taxa from the Great Barrier Reef (GBR) and aquaria, the study resolved hypoxic thresholds for oxyregulation for the first time for key coral reef‐forming species, extending beyond other observations that had captured time‐dependent limits of survival to specific low O_2_ concentrations (e.g., Alderdice et al., 2021; Johnson, Scott, et al., 2021; Johnson, Swaminathan, et al., 2021).

Previous studies have utilised a range of methods to parameterise the change in O_2_ consumption as a function of declining O_2_ availability based on HRC analysis (e.g., Rutherford & Thuesen, 2005; Ultsch & Regan, 2019; Zhang & Farrell, 2022). This includes the ‘regulation profile’ method proposed by Cobbs and Alexander (2018), which derives broad descriptive statistics of an organisms oxyregulatory capacity. For example, the extent of total positive regulation (T_pos_, relative units)—or the ‘average’ regulation beyond strict oxyconformity—and the minimum and maximum regulation capacity (P_cmin_/P_cmax_), that is, the *p*O_2_ level (% air saturation) at which taxa exert minimum/maximum regulation effort (Cobbs & Alexander, 2018). The P_cmax_ parameter has also been proposed as an extension of the well‐known critical O_2_ tension parameter, P_crit_ (Cobbs & Alexander, 2018) or P_crit‐max_ (Seibel et al., 2021), which define the lowest *p*O_2_ level (% air sat) at which an organism can maintain a constant VO_2_ (Pontes et al., 2023; Regan et al., 2019; Seibel et al., 2021), that is, the maximum oxyregulation capacity. In this way, P_cmax_ describes the hypoxic threshold for upregulation, where regulation capacity cannot increase past this point. Recent work applying HRCs showed that corals can exhibit moderate capacity for oxyregulation, with substantial variability among coral taxa, and interestingly finding greatest differences in total positive regulation (T_pos_) between species of the same genus, e.g., *Pocillopora damicornis* displaying the lowest average total positive regulation (0.41), compared with *P. acuta* (2.42) (Hughes, Alexander, et al., 2022).

Tropical coral reefs span highly dynamic O_2_ environments, from shallow waters of tidal pools and fringing reefs that regularly endure natural O_2_ depletion, to much deeper reefs (>100 m) threatened by oxygen minimum zones (OMZs; Giomi et al., 2019; Hughes et al., 2020; Nelson & Altieri, 2019). The limited amount of data in the literature suggest that coral taxa exhibit a broad range of O_2_ tolerance and hypoxic thresholds (Alva García et al., 2022; Johnson, Scott, et al., 2021; Johnson, Swaminathan, et al., 2021; Pontes et al., 2023) and regulatory dynamics (Alderdice et al., 2021; Hughes, Alexander, et al., 2022). Yet, it is unclear how these differences reflect inherent tolerance to deoxygenation exposure for any given individual (Deleja et al., 2022) and whether hypoxic thresholds are fine‐tuned by acclimatisation to different environments remains unknown. That said, for corals changes in flow are likely critical to local O_2_ availability whereby colony growth form can define the local flow regime (Hossain & Staples, 2020; Jimenez et al., 2011). Edge environments of corals, in comparison with the centre of colonies, have been found to experience higher flow (Hossain & Staples, 2020), which in turn shapes colony level variance of bacterial communities (Fifer et al., 2022). Given that HRCs in the past typically do not employ standards for acquisition of coral samples, it is plausible that differences in colony sample location—and particularly for complex branching morphologies—will experience very different O_2_ dynamics inside than out—however, whether this results in different hypoxic thresholds for oxyregulation remains untested.

Here we use HRC analysis to test the hypothesis that intra‐colony variation in hypoxic tolerance exists for *Acropora* species common on the GBR, with interior branches exhibiting inherently lower thresholds for hypoxia (and an increased oxyregulatory capacity) compared to ‘exterior’ branches of the same colony. First, we conducted an experiment to analyse whether the process of sampling (i.e., removing fragments) induces stress that may alter O_2_ physiological parameterisation. Second, we aimed to advance current HRC model fitting—and address unresolved key methodological steps to improve confidence in HRC parameter retrieval. To do this, we compared oxyregulatory descriptive statistics extracted from models with multiple‐polynomial degrees (up to 12th order) (Hughes, Alexander, et al., 2022), to the most parsimonious model (i.e., simplest model with fewest parameters), since the biological rationale for the amount of inflexion points necessary in fitting HRCs remains undefined as are the specific biological mechanisms employed by corals during the process of oxyregulation (Hughes, Alexander, et al., 2022). To address these various questions, we examined a range of *Acropora* species—*A. hyacinthus*, *A. intermedia*, and *A. kenti* (formerly *A. tenuis*, Bridge et al., 2023)—as originally sampled by Hughes, Alexander, et al. (2022), as well as *A. loripes*, *A. abrotanoides*, *A*. cf. *microphthalma* and *A. elseyi*, thereby adding insight into inherent inter‐species and intra‐colony variance in oxyregulatory hypoxic thresholds for this key reef‐building genus. Results from this work highlight important considerations for future sampling of corals, including any potential spatial variation in the future susceptibility of coral colonies to deoxygenation events, to further resolve their variable O_2_ physiologies.

## 2 MATERIALS AND METHODS

### 2.1 Coral fragment collection

Coral fragments of ~5–10 cm length were collected using wire cutters from select *Acropora* colonies of branching morphology (Table 1) from Opal Reef (GBR, Australia; 16.220 °S, 145.885 °E), for two experiments. All fieldwork and collections were carried out from the 2nd to the 17th of February 2022, under Permit No. G20/43740.1 (Great Barrier Reef Marine Park Authority). We note that the laboratory set‐up allowed for a maximum of six coral fragments per incubation; therefore, sampling was carried out over multiple days, as further described below.

**TABLE 1:** Properties of *Acropora* colonies with different branching morphology sampled in the field at Opal Reef (GBR, Australia) from the 5th to the 17th of February 2022.

Fragment image		Coral species	Site at opal reef	No. colonies	Sample size	Date sampled	Colony depth (m)	Colony size (m)
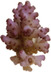	1a	*A. loripes*	Rayban	6	*n* = 6 clipped with CoralClip®	Feb 2, 2022	3.5–4	0.3–1
	1b			6	*n* = 3 clipped, 3 fresh	Feb 8, 2022	3.5–4	
					*n* = 3 clipped, 3 fresh	Feb 9, 2022		
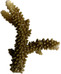	2	*A. abrotanoides*	Mojo	1	O_2_ Logger deployed *n* = 3 interior, 3 exterior	Feb 5, 2022 Feb 6, 2022	2	0.95
					*n* = 3 interior, 3 exterior	Feb 7, 2022		
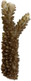	3	*A*. cf. *microphthalma*	Mojo	1	O_2_ Logger deployed *n* = 3 interior, 3 exterior	Feb 7, 2022 Feb 10, 2022	3	1.2
					*n* = 3 interior, 3 exterior	Feb 11, 2022		
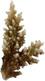	4	*A. elseyi*	Rayban	1	O_2_ Logger deployed *n* = 3 interior, 3 exterior	Feb 12, 2022 Feb 12, 2022	3.5	1.5
					*n* = 3 interior, 3 exterior	Feb 13, 2022		

*Note*: NB: total fragment sample size for interior/exterior of each colony was *n* = 6.

#### 2.1.1 Experiment 1: Fragmentation effects

We first tested for any potential stress induced by physically fragmenting coral, and how recovery time could potentially influence O_2_ drawdown during closed system respirometry. For this, a total of six colonies of *A. loripes*—of varying colour and similar colony size (<1 m) and depth (~4 m)—were selected at Rayban, Opal Reef (Table 1; Lines 1a and 1b) and marked with flagging tape. From each colony, one fragment was removed and immediately secured back onto the reef substrate next to its parent colony location using a CoralClip® (Suggett et al., 2020). After 7‐day postfragmentation recovery, we returned to the same reef location and collected fragments from the CoralClip® (*n* = 3 fragments), on the 8th of February 2022, and again on the 9th of February 2022, as well as taking new fragments from each of the same six colonies (*n* = 3 fragments) on both days (Table 1). All samples (*n* = 6 clipped, *n* = 6 fresh, total) were taken from parent colonies at least 5–10 m apart.

#### 2.1.2 Experiment 2: Interior versus exterior colony variance

To determine whether and how sampling location could potentially affect the O_2_ physiology of the coral, three *Acropora* species (*A. abrotanoides*, *A*. cf. *microphthalma*, and *A. elseyi*) from three colonies (one colony per species) at varying water depth (2–4 m) were selected (Table 1; Lines 2–4). Replicate fragments were removed from each chosen colony, first from the thicket interior (*n* = 3), and then the exterior open branches (*n* = 3), twice over two sampling days (see Table 1 for full sampling schedule).

For both experiments, coral fragments were collected by SCUBA and placed into Ziplock bags for immediate transport to the surface support vessel. Samples were then immediately transported (~60 min) back to a temporary laboratory setup on shore in a 40‐L portable cooler box filled with fresh seawater collected at the site of origin. During transportation, the cooler box lid was left open, corals were shaded from direct sunlight using a black mesh cover, and temperature was monitored regularly using a floating glass thermometer (unbranded). Seawater was constantly aerated using two air‐stones connected to battery‐operated air pumps (Aqua One Battery Air 250). Prior to reaching shore (after ~30 min of travel), 90% of the total seawater in the cooler box was exchanged for fresh seawater (also collected from site)—without exposing the coral fragments to air—before being immediately transported from the boat for respirometry assessment. Dissolved oxygen (DO) levels of the seawater were tested again immediately after transportation from the boat to the onshore laboratory using a robust oxygen probe (OXROB10‐SUB; PyroScience, Germany) connected to an oxygen data metre (FireSting‐O2; PyroScience GmbH, Germany)—remaining above 87% air sat.

### 2.2 Hypoxia response curve (HRC) measurements

Following transportation from Opal Reef (as described in Section 2.1) and prior to incubation, each individual coral fragment was inspected and carefully cleaned of any additional debris (e.g., detachable algae and crabs) using forceps and a soft‐bristled toothbrush (as per Hughes, Alexander, et al., 2022), before being placed in individual respirometry chambers filled with fresh, aerated seawater from the sampling site. Chambers were sealable glass jars (400 mL), each with a hinged wire clasp gas‐tight lid, containing a plastic mesh‐platform upon which the fragment was placed, allowing space beneath for free rotation of a 30 × 7 mm magnetic stirring bar (PTFE, ROWE Scientific Pty Ltd). Each chamber was fitted with an optical O_2_ sensor spot (OXSP5; PyroScience) fixed on the inside of the glass with silicon glue (SPGLUE, Elastosil E43, WACKER, US), for contactless sensor readout via a fibre‐optic O_2_ probe (SPFIB‐BARE; PyroScience) mounted on the transparent chamber. Each probe was connected to a fibre‐optic O_2_ metre (FireSting‐O2, PyroScience GmbH) to continuously monitor the dissolved O_2_ content (% air sat) of the chamber. All O_2_ sensor spots were calibrated against 100% air‐saturated seawater and a 0% O_2_ solution prepared by adding sodium sulphite (Na_2_SO_3_) to de‐mineralised water, prior to incubations.

Corals were acclimated for at least 60 min in their individual chambers, which were left unsealed and constantly aerated by an air‐stone connected to an air pump (Marina 200 Aquarium Air Pump), ensuring DO was maintained at >90% of air saturation during this time as per Hughes, Alexander, et al. (2022). Chambers were three‐quarters submerged in a water bath maintained at 27 ± 0.5°C (corresponding to in situ temperature at the time of sample collection) by a heating immersion circulator (EH, JULABO, Julabo USA, Inc.), and positioned on top of a multi‐station stirring plate (iStir HP 10 M, Neuation Technologies Pty Ltd) to magnetically stir the individual stir bars (~500 rpm). Following acclimation, at least 90% of the seawater in each individual chamber was exchanged, temperature‐adjusted to 27°C by a 150 W bar heater (Aqua One Submersible Glass Heater, 230 V/50 Hz) and aerated with a water pump (Aqua Pro Tabeltop Feature Pump, AP200LV). To avoid exposing the coral fragment to air during this exchange, one end of silicon tubing was connected to the water pump in the fresh seawater, with the other end positioned inside the bottom of the chamber to turnover old with fresh seawater. The chambers were then sealed with the wire clasp gas‐tight lid whilst completely submerged, ensuring no trapping of air bubbles inside.

Closed‐system respirometry was used to measure HRCs (Hughes, Alexander, et al., 2022; Killen et al., 2021). The closed chambers were fully submerged in the heated water bath during incubation, with the entire bath surrounded by purpose cut black‐out cloth and boards to emulate dark conditions of the natural diel cycle (Alderdice et al., 2021). Starting with DO levels as close to 100% of air saturation as possible, drawdown rates of the ambient O_2_ (*p*O_2_) were monitored as coral fragments respired. Dissolved O_2_ content was measured every 60 s, and incubations were terminated after reaching 0% air saturation (or at least <2% air sat during time limitations), over a period of ~6–12 h. Ancillary microbial respiration in the seawater was also measured via supplementary control incubations containing seawater only from the corresponding coral sampling site. In addition, volumes of seawater displacement were determined to normalise individual fragment size to chamber capacity. O_2_ consumption rates from the seawater controls and the volumes displaced by the corals were subtracted from the rates measured of the individual corresponding coral fragments. Mean O_2_ consumption rates for each coral fragment were then calculated, per hour (VO_2_, mg h^−1^).

### 2.3 In situ O_2_ logger deployment

Prior to each sampling event (as described above in Section 2.1), two optical O_2_ sensor data loggers (AquapHOx‐L‐O2; PyroScience GmbH) were deployed to measure the corresponding ambient O_2_ and temperature (with a logging interval set at 60 s) in the *Acropora* colonies, from where the interior versus exterior coral samples were taken (Table 1; Lines 2–4). Due to constraints surrounding boat scheduling, the O_2_ loggers were deployed 1 day prior to the first coral fragment collection in *A. abrotanoides* and *A. elseyi*, and 1.5 days prior to first sampling in *A*. cf. *microphthalma*. A robust O_2_ probe (OXROB10‐SUB; PyroScience) was attached to the SUB‐connector optical port of each data logger, and the tips of these sensors were positioned in the same areas of the colonies from where the fragments were taken for respirometry assessment, that is, (1) in the interior thicket, and (2) in the exterior open branches. The sensors were cable‐tied in position, deployed for 2–3 days at a time, measuring DO (mg O_2_ L^−1^) at 60‐s intervals. The main bodies of the loggers were secured to lead diving weights and positioned on the reef substrate next to the chosen colony, additionally measuring the ambient seawater temperature (°C). The AquapHOx‐L‐O2 loggers were calibrated against 100% air‐saturated seawater and a 0% O_2_ solution prior to deployment.

### 2.4 Statistical analysis

All statistical analyses were carried out in RStudio version 4.1.0, Build 446 (R Core Team, 2021) with base packages: knitr v. 1.33 (Xie, 2014, 2015, 2021), rmarkdown v. 2.20 (Allaire et al., 2023; Xie et al., 2018, 2020), and tidyverse v. 2.0.0 (Wickham et al., 2019).

#### 2.4.1 Hypoxia response curve modelling

**FIGURE 1:**
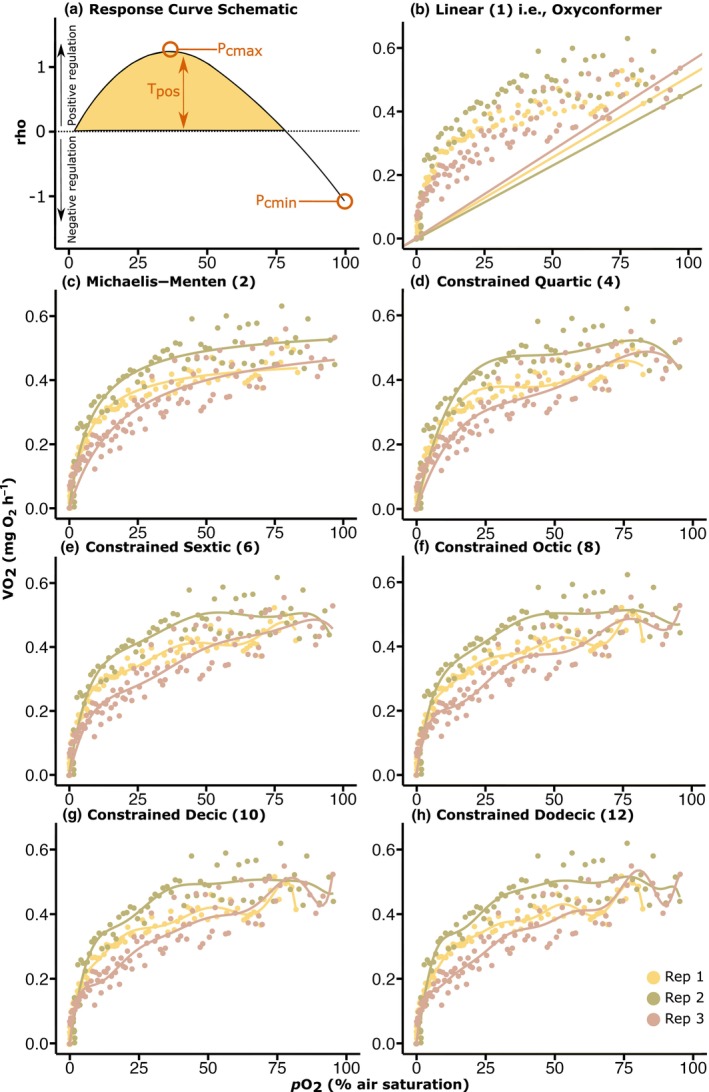
(a) Schematic diagram outlining points of extraction for hypoxia response curve (HRC) parameters (T_pos_, P_cmax_ and P_cmin_) using the rho(pO_2_) equation (Equation 1), in addition to (b–h) models of varying polynomial degrees (1–12th order) fit to replicates of HRC data sets of model species *Acropora kenti* (*n* = 3) (from Hughes, Alexander, et al., 2022). Replicates indicated by colour, and individual model fits (per rep) by lines.

An initial assessment of model fit via Akaike Information Criterion (AIC) for oxyregulation parameter extraction (e.g., T_pos_, see Figure 1a) was made using data previously collected and analysed by Hughes, Alexander, et al. (2022) using the ‘regulation profile’ method (Cobbs & Alexander, 2018). This ensured data retrieved from our study was comparable to that from species of *Acropora* previously analysed. Models of varying polynomial orders—increasing from a linear (1st degree) through to a constrained dodecic (12th degree) polynomial—were fit to three sets of *Acropora* HRC data (*n* = 3)—*A. hyacinthus*, *A. intermedia* and *A. kenti*—previously collected via the same respirometry assay method as for our current study (Hughes, Alexander, et al., 2022). Note that as per (Hughes, Alexander, et al., 2022), an AIC selection of a 1‐parameter linear model fit would suggest strict oxyconformity, that is, no oxyregulation being performed by the coral (similar to ‘Model S1’ as described in Cobbs and Alexander (2018)), whilst any other model selected above a first‐degree polynomial indicates some capacity for oxyregulation. All model fits and plots prepared here used RStudio installed packages ‘drc’ v. 3.0.1 (Ritz et al., 2015) and ‘ggplot2’ (Wickham, 2016).

Following this, oxyregulatory descriptive statistics (T_pos_, P_cmax_ and P_cmin_) were extracted from the parameter estimates of the above model fits using the regulation function rho(pO_2_) (Cobbs & Alexander, 2018):

(1) $$\mathrm{rho}\left({\mathrm{PO}}_{2}\right)=\frac{f\left({\mathrm{PO}}_{2}\right)}{{\mathrm{PO}}_{2}}-f^{\prime }\left({\mathrm{PO}}_{2}\right)$$

Significant differences between the extracted parameters from each HRC fit were tested using a series of independent sample *t*‐tests using RStudio installed package ‘rstatix’ version 0.7.2 (Kassambara, 2023b). Note that the units for T_pos_ are ‘relative’, whilst units for P_cmax_ and P_cmin_ are given as O_2_ concentrations in units of % air saturation. All data for analyses were checked for normality using the Shapiro–Wilk test, and distribution via Q‐Q plots, whilst homogeneity of variance was checked using Levene's test. We used a correlation and regression analysis, using RStudio installed packages ‘car’ (Fox & Weisberg, 2019), ‘ggpubr’ (Kassambara, 2023a), and ‘ggplot2’ (Wickham, 2016), to examine the relationship between the parameters extracted from the original HRCs of *A. hyacinthus*, *A. intermedia*, and *A. kenti* as per (Hughes, Alexander, et al., 2022) and those from the selected polynomial model fit to the same sets of data.

Once the appropriate model was selected (Section 3.1), HRCs generated from the data sets collected during our experimental study were analysed in the same way for *A. loripes*, *A. abrotanoides*, *A*. cf. *microphthalma* and *A. elseyi*. The relevant oxyregulatory descriptive statistics (T_pos_, P_cmax_ and P_cmin_) were then extracted using Equation (1) for comparison. We note that since the selected colonies were independent and from different sites, we did not make inter‐colony comparisons. Instead, we used (i) independent sample *t*‐tests to compare intra‐colony differences for each species (i.e., between the interior vs. exterior thicket samples), and (ii) completed a correlation analysis to compare whether differences between the “average” regulation (i.e., T_pos_) values measured in the interior/exterior coral fragments from the HRCs, corresponded with changes in DO (mg O_2_ L^−1^) measured from the loggers deployed at the same positions (as further described below).

#### 2.4.2 In situ O_2_ logger data analysis

Environmental O_2_ levels (DO, mg O_2_ L^−1^) were evaluated using independent sample *t*‐tests to compare any differences between ambient O_2_ measured via the robust O_2_ probes positioned at the interior thickets and the exterior section of the individual colonies of *A. abrotanoides*, *A*. cf. *microphthalma*, and *A. elseyi*. Additionally, diel cycle information (i.e., sunrise/sunset times) and tidal times (hh:mm:ss) and tidal height (m) data were collected for Opal Reef over the experimental period (BOM, 2023; WillyWeather, 2023), and plotted against the environmental O_2_ and temperature (°C) data collected, to decipher whether the O_2_ profiles corresponded with tidal flushing, and/or diel cycle variance. Furthermore, cumulative histograms were prepared to show accumulative time spent (%) at each level of dissolved O_2_ measured within the individual colonies, to make supplementary intra‐colony comparisons.

## 3 RESULTS

### 3.1 Model analysis and selection: *A. hyacinthus*, *A. intermedia* and *A. kenti*

The linear (1st degree) model was consistently the ‘worst’ fit according to the AIC and Residual Sum of Squares (RSS, i.e., the highest value) for every replicate (*n* = 3) of the three *Acropora* species: *A. hyacinthus*, *A. intermedia* and *A. kenti* (Figure 1b, and Table 2; example species *A. kenti* shown, see Figures S1 and S2 for *A. hyacinthus* and *A. intermedia*). However, and as expected, the 10th‐ or 12th‐order polynomials generally returned the lowest AIC and RSS values (Table 2, Figures S1 and S2), suggesting ‘best’ fit, most likely from the higher number of degrees of freedom, and modelling inflexion points fitting directly through the data points (Figure 1g,h). Interestingly, during this model fit process, one replicate of *A. kenti* selected the Michaelis–Menten (MM) as the best fit function, with the lowest AIC for that group (Figure 1c; Rep 2, AIC −239.72), which similar to (Hughes, Alexander, et al., 2022), was also chosen specifically as the best fit for one replicate. As per (Hughes, Alexander, et al., 2022), a MM fit chosen as the ‘best model’ suggests a continuous regulatory process within the organism.

**TABLE 2:** Mean descriptive statistics and model fit data extracted from models of varying polynomial degrees with varying degrees of freedom (from 1st through to 12th order), fit to hypoxia response curve data sets (*n* = 3) of *Acropora kenti* (data from Hughes, Alexander, et al., 2022); including total positive regulation (T_pos_, relative units), the *p*O_2_ value (% air saturation) at which maximum and minimum regulation (P_cmax_ and P_cmin_) occur, and model fit parameters including Akaike Information Criterion (AIC), and Residual Sum of Squares (RSS) for the model fits (where the lowest values signify the “best fit”).

Model fit	Free parameters	T_pos_ (relative)	P_cmax_ (%)	P_cmin_ (%)	AIC	RSS
Linear	1	NA	NA	NA	−193.02	0.49
Michaelis–Menten	2	0.77	11	94	−281.58	0.17
Constrained Quartic	4	0.73	73	45	−261.13	0.19
Constrained Sextic	6	0.84	69	80	−283.79	0.15
Constrained Octic	8	0.92	34	75	−295.87	0.13
Constrained Decic	10	2.44	64	45	−295.89	0.12
Constrained Dodecic	12	79.42	33	30	−296.60	0.12
Hughes, Alexander, et al. (2022)	12	1.55	17	49	NA	NA

**FIGURE 2:**
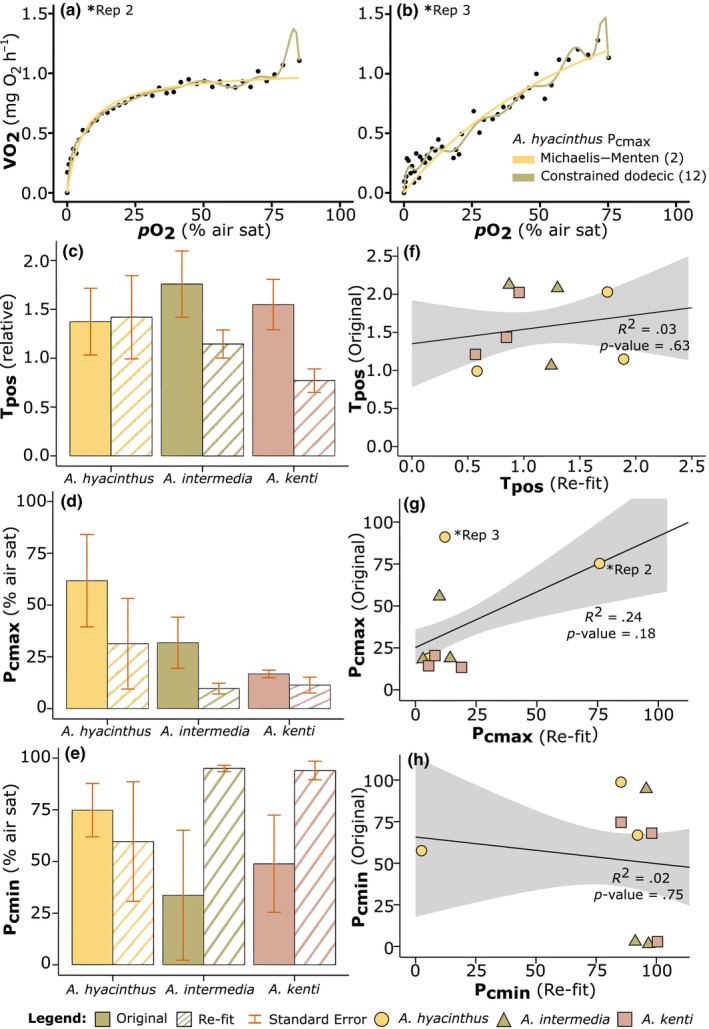
(a) and (b) *Acropora hyacinthus* hypoxia response curve (HRC) data set Rep 2 and Rep 3, fit with 2‐parameter Michaelis–Menten (MM) function (yellow line), and 12th‐order constrained dodecic model (green line). Reps 2 and 3 also annotated on (g) P_cmax_ scatterplot | (c–e) Extracted parameters: T_pos_ (relative units), P_cmax_ and P_cmin_ (% air saturation) from HRC data as analysed by Hughes, Alexander, et al. (2022) i.e., ‘Original’ (solid colour), and re‐analysed here, that is, ‘Re‐fit’ (striped colour) using the MM model, of *Acropora* species (*n* = 3): *A. hyacinthus*, *A. intermedia*, and *A. kenti*. Error bars are calculated standard error. | (f–h) correlation analyses of the same extracted data: T_pos_, P_cmax_, and P_cmin_, from original fits (*y*‐axes), and from re‐fit curves using MM model (*x*‐axes). Linear *R* ^2^ values calculated across averages (shown as the solid black line), and Pearson's Correlation Coefficients (not shown): T_pos_ = 0.19, P_cmax_ = 0.49, and P_cmin_ = 0.12, and the significance of the correlation coefficients (where *p*‐value < .05 is considered significant, shown). Shading indicates the 90% confidence interval.

Extracted T_pos_, P_cmax_ and P_cmin_ values from models with fewer degrees of freedom (2nd through to 8th order), for all *Acropora* species, were all within the same order of magnitude as per previously extracted values (Table 2). For example, mean T_pos_ for *A. kenti* extracted from the re‐fit using the MM model (T_pos_ = 0.77) was half that of the original extracted parameter from the fit by Hughes, Alexander, et al. (2022) (T_pos_ = 1.55; Table 2, and Figure 2a), but not significantly lower (*t*_(4)_ = 2.74, *p* > .05). In fact, there were no significant differences between any of the extracted parameters (T_pos_, P_cmax_, P_cmin_), between the original and MM re‐fit models, for any of the three *Acropora* species (*t*‐tests; Table S1). In general, the extracted values above the 10th‐order polynomial fits were much higher and did not align with the previously reported numbers (Table 2), suggesting an overfit of the data with these selected models.

Additionally, we performed a correlation analysis of the independent variables extracted from HRCs (T_pos_, P_cmax_, P_cmin_), based on those retrieved from (Hughes, Alexander, et al., 2022) versus our MM model re‐fit (Figure 2f–h). Of the three *Acropora* species, an especially strong correlation was evident between the parameters extracted for *A. kenti*. Specifically, for P_cmax_ (*r*_(2)_ = .90, *p* = .29) with a high coefficient of determination (*R* ^2^) of .81, and no significant difference between the means of the two groups—as well as T_pos_, with a strong positive correlation, *r*_(2)_ = .87, *p* = .33, and an *R* ^2^ of .76 (not shown). However, across all three species, there were no significant differences between the means of the two groups (*p*‐value > .05), and there was a moderate positive relationship between the T_pos_ values (*r*_(7)_ = .19, *p* = .63) and P_cmax_ (*r*_(7)_ = .49, *p* = .18), and a weak negative relationship between the values for P_cmin_ (*r*_(7)_ = .12, *p* = .75; Figure 2f–h). See also Figure 2g where *A. hyacinthus* Rep 2 and Rep 3 have been annotated on the correlation analysis scatterplots.

Since parameters extracted from the models with fewer degrees of freedom were not significantly different from the original data sets, and the biological rationale for the number of inflexion points required to fit HRCs remains unclear, we selected the most parsimonious model to minimise the degrees of freedom. The Michaelis–Menten function—a 2‐parameter kinetics‐type function more commonly seen applied to enzyme reactions (Roskoski, 2015; Wood, 2018)—best captured the shape and nature of the data, across all three *Acropora* data sets analysed, with a more conservative approach using fewer free parameters. Following this selection, the MM fit was then applied to all HRCs for *Acropora* data sets collected during our experimental study, and the relevant oxyregulatory descriptive statistics (T_pos_, P_cmax_, P_cmin_) were extracted and analysed.

### 3.2 Experiment 1—Fragmentation effects: *A. Loripes*

**FIGURE 3:**
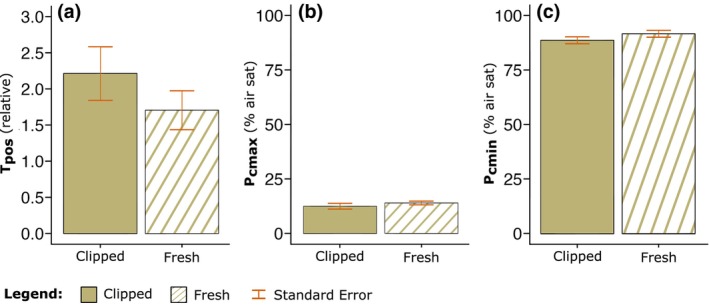
Comparison of the extracted parameters: (a) T_pos_ (relative units), (b) P_cmax_, and (c) P_cmin_ (% air saturation) from hypoxia response curve data of replicates (*n* = 6) of *Acropora loripes*. Plots compare extracted parameters from fragments clipped to the reef substrate for 7 days using the CoralClip® (solid colour), and extractions from samples freshly fragged (striped colour), fit with the selected Michaelis–Menten model. Error bars are calculated standard error.

The mean drawdown rate from 100% to 0% *p*O_2_ (% air sat) of coral fragments left to recover for 7 days (hereafter referred to as ‘clipped’) was 1.36 times faster than that for freshly fragmented corals, but not significantly lower (*t*_(5)_ = 2.57, *p* = .28; Table S2A), at 2.85 h ± SE 0.47, compared to 3.86 h ± SE 0.70, respectively. Total positive regulation was also higher for the clipped fragment (T_pos_ = 2.21 ± SE 0.37) compared to the fresh fragment (T_pos_ = 1.71 ± SE 0.27; Figure 3a), but again no significant difference between the two groups (*t*_(10)_ = 1.11, *p* = .29). In general, values for both P_cmax_ and P_cmin_—that is, the *p*O_2_ level (% air sat) at which maximum/minimum regulation occurred—were lower in the clipped fragments, compared with the freshly fragmented corals. Specifically, P_cmax_ for both the clipped and freshly fragmented corals performed their maximum regulation effort at relatively low *p*O_2_, below 14% air saturation, with no difference between treatments (*t*_(10)_ = 0.95, *p* = .37; Figure 3b, Table S2A). Corals in both treatments exerted minimum regulation effort (P_cmin_) at similar *p*O_2_, 89.17% ± SE 1.60, and 92.17% ± SE 1.58 (Figure 3c), for the clipped and fresh fragments, respectively. Collectively, these data confirmed that mean O_2_ drawdown rates and oxyregulatory capacity remained the same whether corals were freshly fragmented or were provided with 7 days recovery post fragmentation.

### 3.3 O_2_ logger data: *A. abrotanoides*, *A.* cf. *microphthalma*, and *A. elseyi*

**FIGURE 4:**
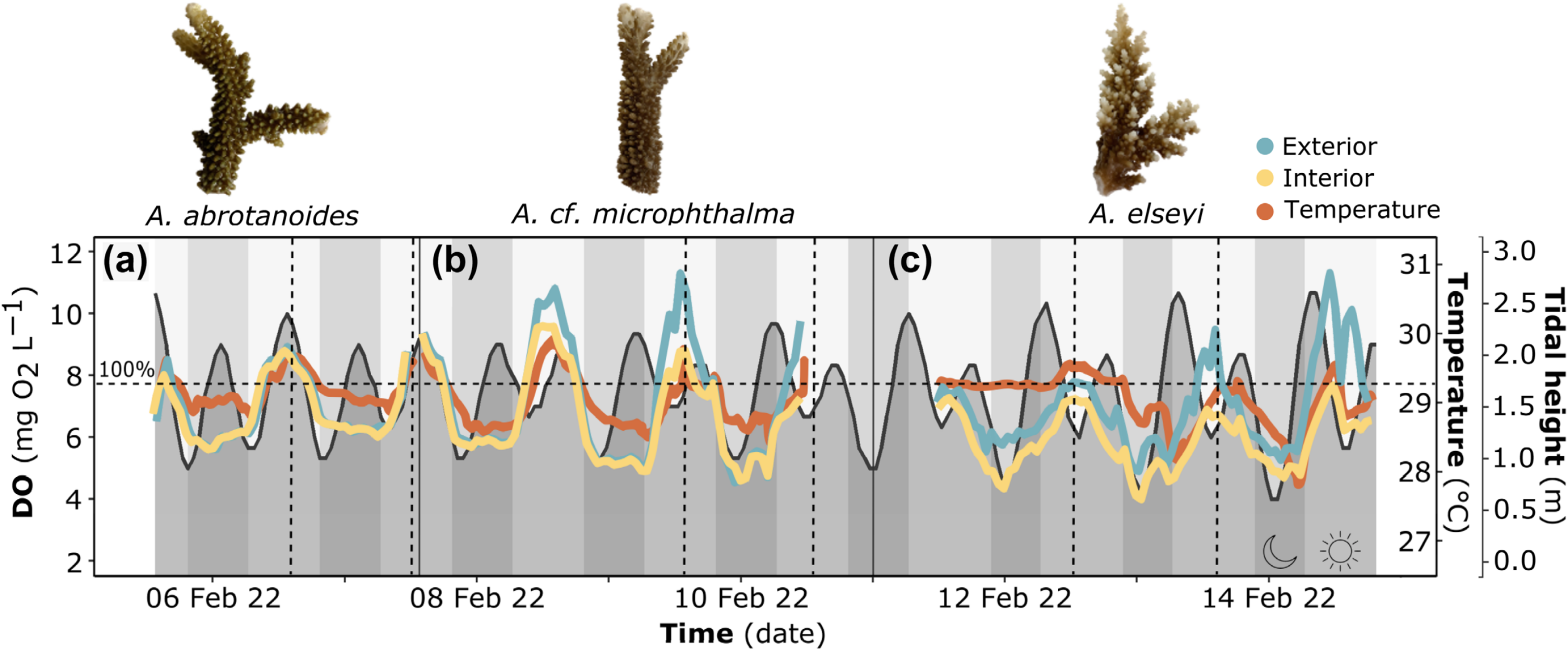
Dissolved oxygen (DO) content (mg O_2_ L^−1^) displayed on the left *y*‐axis was measured at the interior (yellow) and exterior (blue) sections of the three branching *Acropora* colonies; panels (a) *A. abrotanoides*, (b) *A*. cf. *microphthalma*, and (c) *A. elseyi*, respectively. Ambient water temperature (orange, °C) on the inside right *y*‐axis, was also measured by the loggers deployed in the sample colonies, and is coupled with tidal height (dark shaded area with black outline, m) on the outside right *y*‐axis. Daily light cycles are represented by vertical shading and signified by the sun and moon symbols, over time on the *x*‐axis (date). Note that vertical dashed lines indicate fragment sampling times for each *Acropora* colony, carried out over the experimental period (as summarised in Table 1), whilst the horizontal dashed line indicates 100% air sat (i.e., at ~7.67 mg O_2_ L^−1^).

For the three *Acropora* species at Opal Reef, seawater temperature and overall O_2_ profiles appeared to reflect diel cycles (e.g., time of day) more than tidal height (Figure 4). This resulted in all examined coral colonies spending 47% of time between 5.50 and 6.50 mg O_2_ L^−1^ (Figure S3). Note that 100% air saturation here corresponds to 7.67 mg L^−1^, calculated at 1013 mbar, 35 ppt salinity and the average recorded temperature 29.12°C (Figure 4). Dissolved O_2_ concentrations (DO) measured within the *A. abrotanoides* colony spanned 5.46–9.63 mg L^−1^ (Figure 4a), in comparison with the *A. elseyi* colony, with measurements ranging 3.74–7.81 mg L^−1^ within the interior thickets, and 4.71–13.80 mg L^−1^ for the exterior branches (Figure 4c; Figure S3). At no time over the recorded period, did the DO drop below <2 mg L^−1^ (i.e., a commonly used threshold for hypoxia); however, in the *A. elseyi* colony, 7% of time was spent below 5 mg O_2_ L^−1^ in the interior thickets, compared to <1% in the exterior (Figure S3). Indeed, the greatest difference in DO (mg O_2_ L^−1^) measured between the interior and exterior of the *Acropora* colonies was observed for *A. elseyi* (*t*_(11620)_ = −50.55, *p* < .001; Table S3) consistently over most tidal and diel changes (Figure 4c). Whilst DO of the *A*. cf. *microphthalma* colony was also significantly higher for the exterior than interior (*t*_(8400)_ = −10.92, *p* < .00), this trend was generally only seen during the middle of the day, which aligned with fragment sampling time (Figure 4b). Overall, DO differences between inner and outer colony locations were small, however, it is evident that DO measured at the interior section were on average ~10% lower than the exterior, suggesting intra‐colony‐specific spatial variation in the O_2_ levels of the seawater surrounding coral branches.

### 3.4 Experiment 2—Interior versus exterior colony HRC variance: *A. abrotanoides*, *A.* cf. *microphthalma* and *A. elseyi*

**FIGURE 5:**
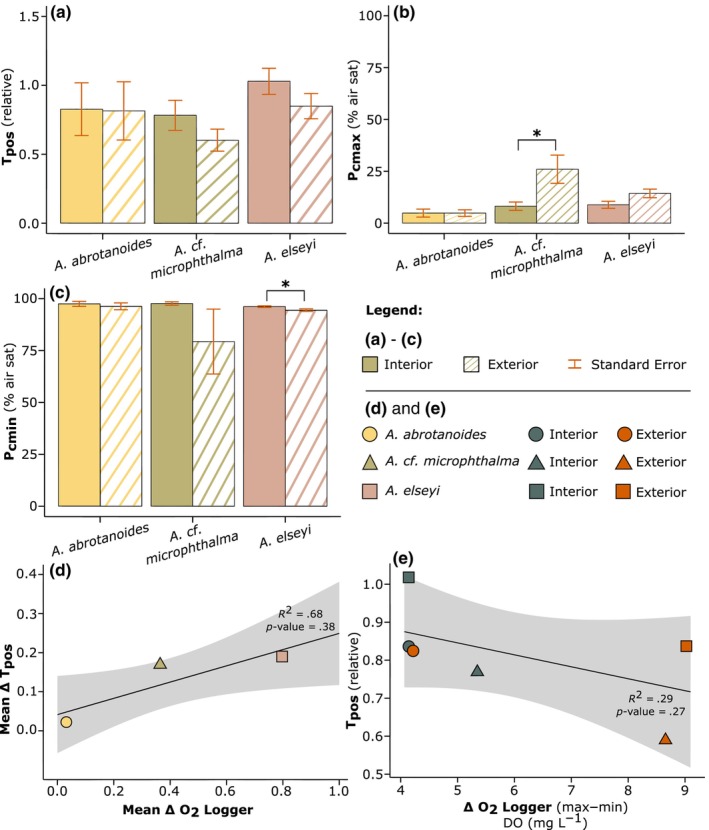
(a–c) Comparison of the extracted parameters: T_pos_ (relative units), P_cmax_, and P_cmin_ (% air saturation) from HRC data of replicates (*n* = 6) of *Acropora* species: *A. abrotanoides*, *A*. cf. *microphthalma*, and *A. elseyi*. Plots compare extracted parameters from fragments taken from the interior section of the colony (solid colour), and fragments from the exterior thickets (striped colour), fit with the selected MM model. Error bars are calculated standard error. Note that significance levels (where *p*‐values < .05 considered significant) are identified by a bold * above relevant data. (d) Correlation analysis between mean interior and exterior delta (Δ) O_2_ logger data (DO, mg L^−1^) and Δ T_pos_ data (relative units), between species. Linear *R* ^2^ was calculated across means (shown as the solid black line), as well as Pearson's Correlation Coefficient = 0.83, and the significance of the correlation coefficient (also shown). (e) Additional correlation analysis between Δ O_2_ logger range (max‐min), and Δ T_pos_ data, between species, with Pearson's Correlation Coefficient = 0.54, and linear *R* ^2^ and significance of correlation coefficient (shown). Shading is 90% confidence interval.

For all three *Acropora* species, the mean (*n* = 6) total positive regulation (T_pos_), and *p*O_2_ (% air sat) at which the minimum regulation effort occurred (P_cmin_), was greater for fragments sampled from the interior than exterior of the coral thickets (Figure 5b,d). Specifically, mean P_cmin_ for *A. elseyi* occurred at a significantly higher *p*O_2_ for the interior (96.17% ± SE 0.40) than the exterior fragments (94.50% ± SE 0.62, Figure 5d, *t*_(10)_ = 2.26, *p* = .05, Table S2B). Except for *A. abrotanoides*, where P_cmax_ was the same on average (P_cmax_ = 4.83%) across interior and exterior samples (Figure 5c), the level at which maximum regulation effort occurred was at a higher *p*O_2_ (% air sat) in the exterior than interior fragments, and significantly higher in *A*. cf. *microphthalma* (at 26% ± SE 6.80), compared to 8.17% ± SE 2.01 (*t*_(10)_ = 2.51, *p* = .03, Figure 5c). Despite the lack of statistical significance between the interior versus exterior T_pos_ values (Figure 5b), differences between the intra‐colony average regulation (T_pos_), were highly consistent with changes in dissolved O_2_ (DO, mg O_2_ L^−1^) measured between the inner and outer colony locations, for all species (Figures 4 and 5). Between the average delta (Δ) O_2_ logger data (DO, mg L^−1^; Figure 4a–c) and the average Δ T_pos_ data (relative units; Figure 5b), there was a positive correlation, albeit non‐significant (*r*_(1)_ = .83, *p* = .38) – with a high coefficient of determination (*R* ^2^ = .68; Figure 5a; Table S2B).

A weak negative correlation (*R* = .54, *R* ^2^ = .29) was also evident between the dynamic range of DO (mg O_2_ L^−1^) measured by the O_2_ loggers (max‐min), and T_pos_, on average (Figure 5e), showing that less DO variation results in higher T_pos_. For example, the maximum range of DO measured in the interior section of the *A. elseyi* colony (4.06 mg L^−1^) – which is the smallest range in DO overall – corresponds to the highest T_pos_ on average (1.03 ± SE 0.09) (Figure 5a,e). Additionally, in both *A. abrotanoides* and *A. elseyi*, O_2_ drawdown rate (% air sat) was slightly faster in the fragments taken from the interior section of the colonies, compared with the exterior thickets, by 103% and 105%, respectively (see Table S4 for mean O_2_ drawdown rates (h)). However, for *A*. cf. *microphthalma*, the O_2_ drawdown rate was 131% faster in the exterior fragments (6.94 h ± SE 0.64), which was significantly more rapid in comparison with fragments sampled from the interior (9.07 h ± SE 0.87, *t*_(5)_ = 2.57, *p* = .01; Table S4). High variance across the sampling—albeit not statistically significant across most comparisons of extracted HRC parameters—indicates that even within intra‐colony sampling (i.e., interior/exterior), fragments are characterised by inherently high variation in respiration.

## 4 DISCUSSION

Coral reefs worldwide are under significant threat from the relatively unstudied process of ocean deoxygenation (Hughes et al., 2020; Pezner et al., 2023; Pontes et al., 2023; Sampaio et al., 2021), in comparison to the well‐known stressors of ocean warming and acidification. Thresholds and performance dynamics with which corals respond to losses of O_2_ remain limited, with only few experimental assessments to date (Alderdice et al., 2021; Alva García et al., 2022; Hughes et al., 2020; Johnson, Scott, et al., 2021; Nelson & Altieri, 2019) since real‐time deoxygenation events are difficult to capture in the field. Such limited knowledge therefore limits current capacity to predict how corals—and in turn coral reefs—will respond to future anthropogenically‐driven declines in reef O_2_ levels. Here, we examined dynamics of O_2_ performance for species of *Acropora* common on the GBR, where previous studies have identified species from this genus to exhibit highly variable responses to both time‐dependent (Alderdice et al., 2021; Johnson, Scott, et al., 2021) and dose‐dependent (Haas et al., 2014) analyses, including HRCs (Hughes, Alexander, et al., 2022). We used initial methodological steps to (a) demonstrate that a simpler model can be used to analyse coral HRCs than previously examined, and (b) establish that immediate fragmenting does not induce added stress during O_2_ drawdown, when compared to fragmenting with a seven‐day recovery‐period. Subsequent measurements of both DO and HRCs for interior versus exterior colony thickets—where the two factors correspond—also exhibited within‐colony variability between species.

### 4.1 Modelling and experimental considerations in describing O_2_ physiology

HRC models have been used to examine how various aquatic organisms including fish (Cobbs & Alexander, 2018; Ultsch & Regan, 2019; Zhang & Farrell, 2022) and corals (Dodds et al., 2007; Hughes, Alexander, et al., 2022) respond to declining O_2_ levels. Experimental HRCs have been analysed with a multitude of oxyregulatory descriptive statistics to parameterise organism O_2_ sensitivity. However, models that fit specific data sets better than others may not necessarily reflect a more accurate model, but rather that the selected model has greater ‘fit propensity’, that is, may fit a larger range of data inadequately, as opposed to a more accurate fit across a smaller range (Falk & Muthukrishna, 2023). Our model analysis demonstrates the challenges of collecting repeatable data without noise, which becomes more apparent when selecting towards a higher polynomial degree (e.g., constrained dodecic, 12th order, Figure 1h). Specifically, greater variance in HRC data results in lower correlation between models (e.g., between the 2nd‐order MM, and 12th order polynomial; see Figure 2b *A. hyacinthus* Rep 3). In contrast, more convergence between the two models, with a higher correlation, is evident when HRC data are less variable (e.g., see Figure 2a *A. hyacinthus* Rep 2). Scattered data for any given HRC demonstrate the high variability in organism respiration, and for corals specifically, the unknown physiological mechanisms occurring between 100% and 0% *p*O_2_ that an organism may employ when DO reaches a certain level. Therefore, we argue that relying on a more conservative model with fewer degrees of freedom (e.g., 2‐parameter MM; Figure 1c) appears more lenient in terms of the challenges of analysing physiological data, ensuring more consistency across biological replicates whilst avoiding overfitting data with higher order models, as evident in Figure 1. However, as our understanding of the physiological mechanisms that the coral holobiont employs during increasingly low levels of O_2_ improves, higher polynomial models may become more appropriate, and can thus be selected based on the known biological mechanisms at play.

### 4.2 O_2_ and HRC dynamics of *Acropora* species

The O_2_ dynamics in coral colonies is strongly affected by the flow of seawater around and within the colonies, which is in turn affected by coral colony size and morphology, including distance between branches and branch diameter (Hossain & Staples, 2020; Hughes et al., 2020). Interactions between flowing seawater and coral structure lead to formation of hydromechanical (Shashar et al., 1996), diffusive (Kühl et al., 1995) and thermal (Jimenez et al., 2008) boundary layers that affect mass and heat transfer between the coral and surrounding seawater. Flow dynamics and boundary layers are not only species‐specific, but dependent on colony‐specific morphological structures (Hossain & Staples, 2020; Jimenez et al., 2011) and coral behavioural features such as ciliary movement induced vortices and local advective flows (Pacherres et al., 2022) and transport between polyps (Bouderlique et al., 2022). We note that the O_2_ budget of tropical corals is partitioned between the coral animal host, its microalgal symbionts, and microbiomes associated within the coral tissue, gastric cavity and coral skeleton (Hughes, Raina, et al., 2022), but the relative importance of these different compartments for observed responses of the coral holobiont to declining O_2_ levels remain unknown. The O_2_ supply of corals can be further modified by fish ventilation, especially under low ambient O_2_ conditions where hyperventilation in gill pumping occurs (Zhang & Farrell, 2022), or by additional respiration from commensal organisms within corals, such as the guard crab *Trapezia* (McKeon & Moore, 2014).

Here, we recorded subtle differences in the ambient DO (mg O_2_ L^−1^) levels, measured at the interior and exterior sections of the three selected *Acropora* colonies for experimental sampling: *A. abrotanoides*, *A*. cf. *microphthalma* and *A. elseyi*. Whilst we were unable to quantitatively resolve differences in factors that may have impacted flow penetration into the colonies examined here, it is intriguing that the greatest difference in intra‐colony recorded DO occurred in *A. elseyi* with an apparent more tightly formed thicket colony structure (Figure 4c and Table 1, Line 4). In contrast, the smallest difference was recorded between interior and exterior branches in *A. abrotanoides*—that exhibits a relatively more spaced‐out colony geometry (Figure 4a and Table 1, Line 2). This is further amplified by the weak but positive correlation (*R* ^2^ = .68, Pearson's Correlation Coefficient = 0.83, see Figure 5d), albeit statistically insignificant (*p*‐value > .05), between the ∆ interior versus exterior T_pos_ values measured in all three *Acropora* species, and the ∆ interior/exterior DO content (mg O_2_ L^−1^), indicating that differences between the intra‐colony average regulation, were highly consistent with changes in DO.

We show a range of highly variable hypoxic thresholds for oxyregulation, with total positive regulation capacity varying from 0.60 to 1.03 (T_pos_, relative) across species, which aligns with the high variation in hypoxic tolerance seen previously in *Acropora* species (Deleja et al., 2022; Hughes, Alexander, et al., 2022; Pontes et al., 2023). For example, *A. cervicornis* had the second lowest *p*O_2_ crit (critical oxygen partial pressure) of 2.22 mg O_2_ L^−1^ (i.e., 7 ± 1 kPa; Pontes et al., 2023), in a study out of six species of Caribbean scleractinian corals (Pontes et al., 2023). A lower *p*O_2_ crit (‘P_crit_’) is suggested to be an advantageous characteristic for surviving low O_2_ periods (Nilsson & Östlund‐Nilsson, 2005; Pontes et al., 2023) – similarly to the low P_cmax_ values ascertained here. On average, *A. abrotanoides* exerted maximum regulation effort at lower *p*O_2_ (% air sat) values (i.e., under low ambient DO) in comparison to all other corals (4.83% ± SE 1.24), for both interior and exterior fragments (Figure 5b; Table S2B), with the highest P_cmax_ value recorded in this study at 26% air sat (*A*. cf. *microphthalma* exterior; Figure 5c). These P_cmax_ values are lower than the lowest observed on average across all coral taxa as analysed by (Hughes, Alexander, et al., 2022) at around 30% air sat—and within a similar range to those noted for coral reef fishes ~10–25% air sat (measured as P_crit_) (Nilsson & Östlund‐Nilsson, 2005). However, we acknowledge that the more conservative approach to modelling adopted here (with fewer degrees of freedom) may account for the lower values seen across descriptive statistics. Nonetheless, *Acropora* therefore appears to be a highly dynamic species, across both hypoxic landscapes (Alderdice et al., 2021; Deleja et al., 2022; Haas et al., 2014; Hughes, Alexander, et al., 2022; Pontes et al., 2023), and additionally in thermal tolerance to heat stress (Alderdice et al., 2022; Hoogenboom et al., 2017; Nielsen et al., 2022).

Whilst the hypoxia threshold of <2 mg O_2_ L^−1^ (or ~25% air sat) is widely cited (Altieri et al., 2021; Hughes et al., 2020; Hughes, Alexander, et al., 2022; Rabalais et al., 2001; Vaquer‐Sunyer & Duarte, 2008)—and although the specific threshold for *Acropora* species is unclear—a general universal threshold may not prove useful simply due to the high variability in sampling (e.g., T_pos_ as seen here), including across other experimental studies. For example, a recent study by Johnson, Scott, et al. (2021) recorded lethal thresholds for the Caribbean coral *Acropora cervicornis* after just 1 day of exposure at 1 mg O_2_ L^−1^, in comparison with the highly variable hypoxia thresholds for upregulation recorded here i.e., P_cmax_ ranging from 4.83% to 26% air sat (or ~0.38 to 2.03 mg O_2_ L^−1^; Figure 5). No periods of potential hypoxia were recorded during logger deployment across any of the *Acropora* colonies, where DO did not drop below 3.5 mg L^−1^ (Figure 4a–c). However, it is important to note here that O_2_ concentrations within the diffusive boundary layer are likely considerably lower at night even within the colony, in comparison with O_2_ levels measured in the water column (Kühl et al., 1995; Shashar et al., 1993, 1996).

Notably, what we have shown is the DO span these *Acropora* species can tolerate, relative to their average regulation (T_pos_) characteristics—where different range exposures over the interior and exterior colony, result in different thresholds of T_pos_. Although weak, there is a negative correlation between the range (∆ max–min interior and exterior) of DO (mg O_2_ L^−1^), and the mean T_pos_, where lower variation in DO corresponds to a higher T_pos_ (Figure 5e). Even with the lowest range in DO overall, *A. abrotanoides* still has a higher T_pos_ on average (0.82 ± SE 0.20) than *A*. cf. *microphthalma* (0.69 ± SE 0.09; Figure 5a) which has a greater dynamic range in DO, across both the interior and exterior colony (Figures 4 and 5e). Additionally, *A. elseyi* had the highest T_pos_ on average, measured at the interior fragments specifically, but also across all three *Acropora* species (1.03 ± SE 0.09), suggesting the greatest ‘average’ regulation across the colonies sampled.

The dynamic range of DO, and extremely high variability in regulation across intra‐colony sampling seen here, with high standard error between replicates (n = 6) for all species, would suggest that even within location sampling (i.e., between the interior and exterior fragments), there is high variation in the respiration rates of corals, which affects within‐colony oxyregulatory capacity. Therefore, among other sampling considerations—e.g., fragment size and sampling time, for heat stress assays (Nielsen et al., 2022)—the outcomes from our methodological findings confirm that, (i) having multiple replicates is essential for producing repeatable data, (ii) although coral respiration rates are highly variable, fragment collection does not appear to induce additional stress to alter O_2_ physiological parameterisation, and (iii) albeit ‘non‐significant’ (likely due to the large standard error across sampling) there does seem to be intra‐colony O_2_ spatial variance, which would be amplified further, without specific location sample acquisition. Additionally, conservative parameterisation of HRC model fitting can yield comparative oxyregulatory statistics to models with a higher number of polynomial degrees, without over‐fitting inflexion points of unknown biological origin. Our data add insight into coral HRC analysis, expanding on the inventories of hypoxic thresholds for upregulation and oxyregulatory capacity for the key coral reef‐building species *Acropora*—including intra‐colony spatial O_2_ variation—as well as expanding on considerations for future fragment sampling collection. Ocean deoxygenation is an emergent threat to coral reefs worldwide, and therefore must be a consideration in future studies in conjunction with the effects of other well‐studied stressors under climate change (e.g., ocean warming and acidification), including oxyregulatory capacity of corals under warming oceans, since increased temperatures increases biological O_2_ demand (Alderdice et al., 2022; Keeling et al., 2010; Pezner et al., 2023).

## AUTHOR CONTRIBUTIONS

**Nicole J. Dilernia:** Conceptualization (lead); data curation (lead); formal analysis (lead); investigation (lead); methodology (lead); resources (lead); software (lead); validation (lead); visualization (lead); writing – original draft (lead); writing – review and editing (equal). **Stephen Woodcock:** Methodology (supporting); software (equal); writing – review and editing (supporting). **Emma F. Camp:** Conceptualization (supporting); methodology (supporting); project administration (equal); supervision (equal); writing – review and editing (equal). **David J. Hughes:** Conceptualization (supporting); methodology (supporting); supervision (equal); writing – review and editing (supporting). **Michael Kühl:** Resources (supporting); supervision (equal); writing – review and editing (supporting). **David J. Suggett:** Conceptualization (supporting); investigation (supporting); methodology (supporting); project administration (lead); supervision (equal); validation (supporting); writing – review and editing (supporting).

## FUNDING INFORMATION

This research is supported by an Australian Government Research Training Program Scholarship (N.J.D.) and an Australian Research Council Discovery Project (DP230100210) (awarded to: D.J.S., E.F.C., and M.K.). M.K. also acknowledges additional support from the Gordon and Betty Moore Foundation (grant no. GBMF9206; https://doi.org/10.37807/GBMF9206).

## CONFLICT OF INTEREST STATEMENT

No competing interests declared.

## Supporting information


Figure S1.

## Data Availability

The data and code that support the findings of this study are openly available in Dryad (https://doi.org/10.5061/dryad.gtht76ht3).
