# Supplementary material for: Intra‐colony spatial variance of oxyregulation and hypoxic thresholds for key Acropora coral species
Source: Ecol Evol. 2024 Mar 5;14(3):e11100. doi: 10.1002/ece3.11100 (PMC10914553; doi:10.1002/ece3.11100)
Supplement: Supplementary file 1 — Figure S1. [file ECE3-14-e11100-s001.docx]

**Appendix**

**Figure S1**: (A) to (G) polynomial models of varying degrees (1-12^th^ order) fit to replicates of hypoxia response curve datasets of model species *Acropora hyacinthus* (n=3) (from Hughes et al. 2022b), and model fit parameters including Akaike Information Criterion (AIC), and Residual Sum of Squares (RSS), where the lowest values signify the “best fit”. Replicates indicated by colour, and individual model fits (per rep) by lines.


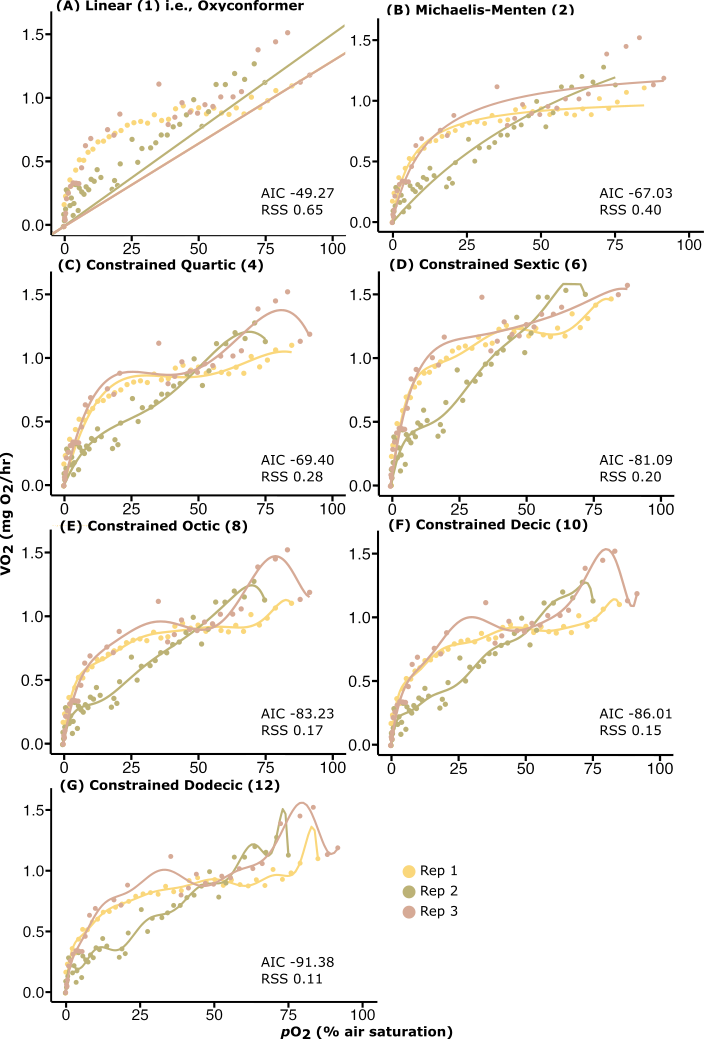


**Figure S2**: (A) to (G) polynomial models of varying degrees (1-12^th^ order) fit to replicates of hypoxia response curve datasets of model species *Acropora intermedia* (n=3) (from Hughes et al. 2022b), and model fit parameters including Akaike Information Criterion (AIC), and Residual Sum of Squares (RSS), where the lowest values signify the “best fit”. Replicates indicated by colour, and individual model fits (per rep) by lines.


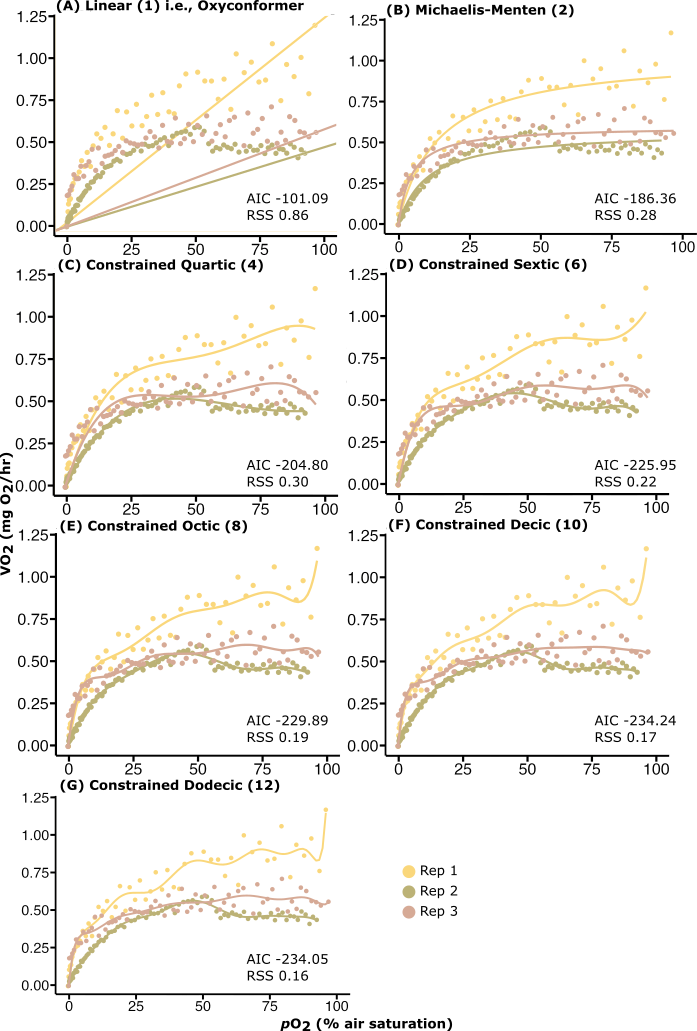


| **Table S1**: Comparison of the mean extracted parameters (T_pos_, P_cmax_ and P_cmin_), from three sets of *Acropora* hypoxia response curve datasets, originally analysed by Hughes et al. (2022b) i.e., “original”, and re-analysed here i.e., “re-fit” using the selected Michaelis-Menten model, for: *Acropora hyacinthus*, *Acropora intermedia*, and *Acropora kenti*. Mean and standard error (*SE*, n=3) for average parameters have been reported. Independent sample T-test results are also reported for extracted parameters for each species, to identify differences between the original and re-fit model extractions. Note that p-values < 0.05 are considered significant and are identified in **bold text*.** Degrees of freedom is “*d.f*.”. | | | | | |
| --- | --- | --- | --- | --- | --- |
| **Coral Species** | **Model fit** |  | **T_pos_**  **(relative)** | **P_cmax_**  **(% air sat)** | **P_cmin_**  **(% air sat)** |
| ***A. hyacinthus*** | Original | Mean  *SE* | 1.37  0.34 | 61.77  22.30 | 74.83  12.93 |
|  | Re-fit | Mean  *SE* | 1.42  0.43 | 31.33  21.88 | 59.67  28.90 |
|  | *T-test* | *t-statistic* | -0.08 | 0.97 | 0.48 |
|  | *d.f. = 4* | *p-value* | 0.94 | 0.39 | 0.66 |
| ***A. intermedia*** | Original | Mean  *SE* | 1.76  0.34 | 31.80  12.35 | 33.67  31.42 |
|  | Re-fit | Mean  *SE* | 1.14  0.14 | 9.67  2.60 | 95.00  1.53 |
|  | *T-test* | *t-statistic* | 1.66 | 1.75 | -1.95 |
|  | *d.f. = 4* | *p-value* | 0.17 | 0.15 | 0.12 |
| ***A. kenti*** | Original | Mean  *SE* | 1.55  0.26 | 16.77  1.81 | 48.93  23.51 |
|  | Re-fit | Mean  *SE* | 0.77  0.12 | 11.33  3.84 | 94.00  4.51 |
|  | *T-test* | *t-statistic* | 2.74 | 1.28 | -1.88 |
|  | *d.f. = 4* | *p-value* | 0.05 | 0.27 | 0.13 |

| **Table S2**: Comparison of the mean extracted parameters (T_pos_, P_cmax_ and P_cmin_), from hypoxia response curve (HRC) datasets from (A) Experiment 1: fragmentation effects testing HRC datasets fit to *A. loripes*, and (B) Experiment 2: interior versus exterior colony variance of the three experimental colonies: *A. abrotanoides*, *A. cf. microphthalma*, and *A. elseyi*, all analysed using the selected Michaelis-Menten model. Mean and standard error (*SE*, n=6) for average parameters have been reported. Independent sample T-test results are also reported for extracted parameters for each species, to identify differences between the clipped fragments allowed 7-days post fragmentation recovery, and freshly fragged corals (Experiment 1), and differences between the interior and exterior of the colony (Experiment 2). Note that p-values < 0.05 are considered significant and are identified in **bold text*.** Degrees of freedom is “*d.f.”.* | | | | | |
| --- | --- | --- | --- | --- | --- |
| **Coral Species** | **Fragment Type** | | **T_pos_**  **(relative)** | **P_cmax_**  **(% air sat)** | **P_cmin_**  **(% air sat)** |
| 1. **Experiment 1: fragmentation effects** | | | | | |
| ***A. loripes*** | Clipped Fragment | Mean  *SE* | 2.21  0.37 | 12.50  1.34 | 89.17  1.60 |
|  | Fresh Fragment | Mean  *SE* | 1.71  0.27 | 14.00  0.86 | 92.17  1.58 |
|  | *T-test*  *d.f. = 10* | *t-statistic*  *p-value* | 1.11  0.29 | -0.95  0.37 | -1.33  0.21 |
| 1. **Experiment 2: interior versus exterior colony variance** | | | | | |
| ***A. abrotanoides*** | Interior | Mean  *SE* | 0.83  0.19 | 4.83  19.4 | 97.50  1.20 |
|  | Exterior | Mean  *SE* | 0.81  0.21 | 4.83  1.60 | 96.33  1.65 |
|  | *T-test* | *t-statistic* | 0.04 | 0.00 | 0.57 |
|  | *d.f. = 10* | *p-value* | 0.97 | 1.00 | 0.58 |
| ***A. cf. microphthalma*** | Interior | Mean  *SE* | 0.78  0.11 | 8.17  2.01 | 97.67  0.84 |
|  | Exterior | Mean  *SE* | 0.60  0.08 | 26.00  6.80 | 79.33  15.63 |
|  | *T-test* | *t-statistic* | 1.33 | -2.51 | 1.17 |
|  | *d.f. = 10* | *p-value* | 0.21 | **0.03*** | 0.27 |
| ***A. elseyi*** | Interior | Mean  *SE* | 1.03  0.09 | 8.83  1.70 | 96.17  0.40 |
|  | Exterior | Mean  *SE* | 0.85  0.09 | 14.33  2.06 | 94.50  0.62 |
|  | *T-test* | *t-statistic* | 1.37 | -2.06 | 2.26 |
|  | *d.f. = 10* | *p-value* | 0.20 | 0.07 | **0.05*** |

**Figure S3:** Cumulative time of dissolved oxygen (DO) measured at the interior and exterior sections of the three branching *Acropora* colonies: (A) *A. abrotanoides*, (B) *A. cf. microphthalma*, and (C) *A. elseyi*. Data was collected over the 2-3-day sampling period in February 2022 (as outlined in Table 1). Bars represent the cumulative time (y-axes, %) where the measurements taken at the interior/exterior were within the specific DO (x-axes, mg O_2_ L^-1^) levels, for each colony.


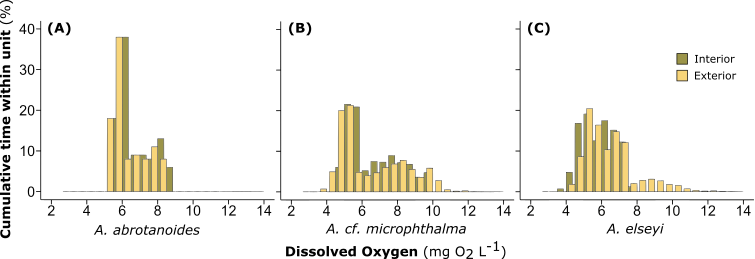


| **Table S3**: Independent sample T-test results comparing oxygen (O_2_) logger data to identify differences between dissolved oxygen content (DO, mg O_2_ L^-1^) measured at the interior section of the colony, compared to the exterior, for the three experimental branching *Acropora* colonies at Opal Reef: *A. abrotanoides*, *A. cf. microphthalma*, and *A. elseyi*. Note that p-values < 0.05 are considered significant and are identified in **bold text*.** Degrees of freedom is *“d.f*.”. | | | |
| --- | --- | --- | --- |
| **Coral Species** | ***d.f.*** | **t-statistic** | **p-value** |
| ***A. abrotanoides*** | 5726 | -1.20 | 0.23 |
| ***A. cf. microphthalma*** | 8400 | -10.92 | **0.00*** |
| ***A. elseyi*** | 11620 | -50.55 | **0.00*** |

| **Table S4**: Comparison of coral drawdown rates (hrs) of the ambient O_2_ (*p*O_2_) calculated from 100 % air saturation, down to 0% air saturation, from the interior and exterior fragments of the three experimental *Acropora* coral species: *A. abrotanoides*, *A. cf. microphthalma*, and *A. elseyi*. Mean and Standard Error (*SE*, n=6) for average consumption rates have been reported. Paired two sample T-test results are also reported to identify differences between the interior and exterior O_2_ drawdown rates per species. Note that p-values < 0.05 are considered significant and are identified in **bold text*.** Degrees of freedom is “*d.f.*”*.* | | | | | |
| --- | --- | --- | --- | --- | --- |
| **Coral Species** | **O_2_ drawdown rate (hrs)** | **Interior** | **Exterior** | **T-test**  **(*d.f*. = 5)** | |
| ***A. abrotanoides*** | Mean | 12.15 | 12.49 | t-statistic | 2.57 |
|  | *SE* | 1.23 | 2.16 | p-value | 0.78 |
| ***A. cf. microphthalma*** | Mean | 9.07 | 6.94 | t-statistic | 2.57 |
|  | *SE* | 0.87 | 0.64 | p-value | **0.01*** |
| ***A. elseyi*** | Mean | 6.65 | 7.01 | t-statistic | 2.57 |
|  | *SE* | 0.60 | 0.40 | p-value | 0.59 |
